# Antroduodenal motility recording identifies characteristic patterns in gastroparesis related to underlying etiology

**DOI:** 10.1111/nmo.14394

**Published:** 2022-05-09

**Authors:** Maartje J. M. Hereijgers, Daniel Keszthelyi, Joanna W. Kruimel, Ad A. M. Masclee, José M. Conchillo

**Affiliations:** ^1^ Division of Gastroenterology and Hepatology Department of Internal Medicine Maastricht University Medical Centre Maastricht The Netherlands

**Keywords:** antroduodenal manometry, gastrointestinal motility, gastroparesis, migrating motor complex

## Abstract

**Background:**

Gastroparesis (GP) is a gastrointestinal disorder associated with significant morbidity and healthcare costs. GP patients form a heterogeneous population with diverse etiology, and treatment is often challenging due to a poorly understood underlying pathophysiology. The aim of the present study was to assess antroduodenal motility patterns among the different GP etiologies.

**Methods:**

We reviewed antroduodenal manometry (ADM) recordings of patients with confirmed GP between 2009 and 2019. ADM measurements were evaluated for fed period duration, number of phase III contractions and migrating motor complexes (MMCs), motility index (MI), and presence of neuropathic patterns.

**Key results:**

A total of 167 GP patients (142 women, median age 45 [31–57]) were included. The following etiologies were identified: idiopathic *n* = 101; post‐surgery *n* = 36; and diabetes *n* = 30. Fed period duration was significantly longer in idiopathic (*p* < 0.01) and diabetic GP patients (*p* < 0.05) compared with post‐surgery GP patients. Furthermore, the number and duration of phase III contractions and the number of MMCs were significantly lower in idiopathic and diabetic patients compared with post‐surgery GP patients (*p* < 0.01). Likewise, absence of MMCs during 6‐h recording was more often observed in idiopathic and diabetes GP patients compared with post‐surgery GP patients (resp. *p* < 0.01 and *p* < 0.05).

**Conclusions and Inferences:**

Antroduodenal motility patterns are different among GP etiologies. A dysmotility spectrum was identified with different patterns ranging from post‐surgery GP to idiopathic and diabetic GP.

## Graphical abstract

Antroduodenal motility patterns are different among etiologies of gastroparesis. A dysmotility spectrum was identified with different patterns ranging from post‐surgery to idiopathic and diabetic gastroparesis.

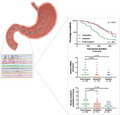

**Key points**

- Antroduodenal motility patterns are different among etiologies of gastroparesis.
- Fed period duration was significantly longer, and the number of phase III contractions and migrating motor complexes was significantly lower in idiopathic and diabetic compared with post‐surgery gastroparesis patients.
- A dysmotility spectrum was identified with different patterns ranging from post‐surgery gastroparesis to idiopathic and diabetic gastroparesis.

## 1 INTRODUCTION

Gastroparesis (GP) is a disorder characterized by delayed gastric emptying (GE) in the absence of mechanical obstruction.^1^ Symptoms include nausea, vomiting, bloating, postprandial fullness, early satiation, and abdominal pain. True epidemiologic data on GP are limited. The estimated prevalence of GP is 13.8/100.000 as documented in general practice records.^2^ It is associated with significant morbidity, leading to higher depression and anxiety scores and reduced quality of life.^3^, ^4^ Furthermore, it has been reported that GP increases the number of hospitalizations resulting in a high medical burden.^5^ There is no validated treatment algorithm for GP, and treatment is mostly tailored on the basis of an individualized approach.^6^ In our center, a stepwise approach is used with dietary adjustments and prokinetics as step 1, enteral feeding by nasoduodenal tube (“gastric rest”) as step 2, and percutaneous endoscopic gastrostomy with jejunal extension as step 3 when step 2 has failed.^7^ The development of more targeted treatment options has stagnated due to poor understanding of the underlying pathophysiology.

Gastroparesis patients form a heterogeneous population with diverse etiology.^8^, ^9^ Approximately, half of all cases is of idiopathic etiology, one‐third of all cases is related to diabetes, and the remaining is related to post‐surgical conditions.^10^, ^11^, ^12^, ^13^ More infrequent etiologies include systemic disorders, such as systemic sclerosis, small fiber neuropathy, and amyloidosis.^14^, ^15^ Common factor of these etiologies is an impaired neuromuscular function leading to delayed GE.^8^, ^16^ In diabetic and idiopathic GP, the neuromuscular dysfunction is thought to be the result of intrinsic neuropathy.^8^ Several studies have reported immune‐modulated loss of the gastric pacemaker cells known as the interstitial cells of Cajal,^17^ loss of enteric nerves, and fibrosis in muscle layers.^18^, ^19^, ^20^, ^21^, ^22^, ^23^, ^24^ This is expected to impair electrical conduction and thereby interfere with gastric coordination. Two studies reported differences in these immunohistological features of the enteric nerve system between diabetic and idiopathic GP patients.^21^, ^23^ In post‐surgery GP, the neuromuscular dysfunction is assumed to be due to extrinsic neuropathy, caused by damage or entrapment of the vagus nerve.^8^

The cornerstone of the diagnosis of GP is based on the measurement of GE. It should be taken into account that GE is poorly associated with symptom severity.^25^ This suggests that in addition to GE, additional diagnostic investigations may be warranted to unravel the distinct histopathological mechanisms mentioned above. A recent study suggested that symptom correlation in GP patients was more closely associated with enteric dysmotility as measured by antroduodenal manometry (ADM) compared with GE.^26^ ADM is a method used in tertiary referral centers to evaluate the gastric and duodenal coordination and activity by recording the overall nature of motility.^27^ The suggested differences in neuromuscular dysfunction between etiologies of GP could potentially lead to differences in gastroduodenal motility patterns. Hypothetically, these etiology‐based differences can be detected by ADM as motility patterns potentially derive from ICC that generate migrating motor complexes (MMCs). To the best of our knowledge, there are no comparative data of ADM parameters between the different etiologies of GP.

The main objective of this study was therefore to assess whether differences in antroduodenal motility patterns are present between the idiopathic, diabetic, or post‐surgery GP when assessed by ADM. We hypothesized that abnormal motility patterns are more often present in idiopathic and diabetic GP (i.e., intrinsic neuropathy) compared with post‐surgery GP (i.e., extrinsic neuropathy).

## 2 MATERIALS AND METHODS

### 2.1 Study population

Between 2009 and 2019 at Maastricht University Medical Centre, ADM data were collected prospectively according to a standardized protocol in a cohort of gastroparesis patients who did not respond to standard treatment of dietary adjustments and prokinetics. GP was defined according to the current criteria^1^: (1) at least one of the following symptoms had to be present: nausea, early satiation, postprandial fullness, vomiting, or bloating; (2) mechanical obstruction had to be excluded by gastroscopy and/or cross‐sectional imaging; and (3) confirmed delayed GE determined by scintigraphy of solids with minimal measuring time of 2 h or ^13^C‐octanoic acid breath test (^13^CBT), or gastric stasis at gastroscopy despite adequate preprocedural fasting. Delayed GE assessed by scintigraphy was generally defined as gastric half emptying time (*T*_1/2_) of at least 100–120 min.^28^, ^29^ This indicated range was chosen due to the fact that measurements were obtained over a longer period of time and from different centers, each with their own protocol and accompanying cutoff values. For ^13^CBT, delayed GE was defined as >130 min on ^13^CBT, which is based on current consensus.^30^ The study was approved by the local ethics committee (METC 16‐4‐130) and was conducted in accordance with the Declaration of Helsinki.

### 2.2 Data collection and protocol

The following data were collected: age, gender, body mass index (BMI), etiology and clinical severity of GP, GE test results, and ADM parameters. Etiology of the gastroparesis was retrieved and categorized as follows: 1. post‐surgery; 2. diabetic; and 3. idiopathic. The clinical severity of GP was categorized into three grades^31^: 1. mild—easily controlled symptoms with maintained body weight on regular diet; 2. compensated—moderate symptoms, partially controlled by medication, nutritional status maintained by dietary adjustment; 3. Decompensated—refractory symptoms, inability to maintain nutritional status via oral intake, and frequent hospital admissions. Given the time frame of this study, GE *T*_1/2_ was assessed by scintigraphy from 2009 to 2016 and by ^13^CBT from 2016 to 2019. To enable the comparison of these different tests, the scintigraphy *T*_1/2_ was converted to ^13^CBT *T*_1/2_ by dividing it with concordance coefficient 0.89.^32^ ADM was performed following standard procedures, after overnight fasting of at least 8 h.^33^ Medication affecting gastrointestinal motility was stopped three days prior to ADM. A solid‐state high‐resolution catheter with 36 transducers spaced at 1‐cm intervals (Unisensor AG) was placed under fluoroscopic control, so that half of the sensors were located distal to the pylorus and the other half in the distal stomach. During the first 30 min, baseline values were recorded in fasting conditions. Subsequently, subjects were given a standardized meal (i.e., one scrambled egg, two slices of white bread with 5 mg of margarine, and 150 ml of water), after which antroduodenal motility was recorded for 6 h.

### 2.3 Outcome parameters

During the first 2 h after meal ingestions, antral and duodenal contraction frequency (contractions/minute), and contraction amplitude (mmHg) were assessed. The motility index (MI) was calculated for this similar timeframe using the formula: $\mathrm{MI}=\ln\left(\left(\mathrm{contraction}\;\mathrm{frequency}*\mathrm{amplitude}\right)+1\right)$.^34^ MI <13.67 was considered as antral hypomotility.^35^ During the full 6 h after meal ingestion, fed period duration was assessed by determining the number of minutes between meal ingestion and the first phase III contraction. Phase III contractions are identified as regular and strong contractions (frequency 2–4/min and amplitude >40 mmHg), lasting at least 2 min migrating from proximal to distal.^34^, ^36^ Most phase III contractions not only originate in the duodenum but can also originate in the antrum. Both localizations were eligible to determine fed period duration. Migrating motor complex is defined by a sequence of phase III contractions followed by phase I, known as a period of quiescence lasting 40–50 min, and phase II contractions with irregular low‐amplitude and random contractions.^27^ In the present study, the number of phase III contractions independently, and the number of MMCs as a full sequence of phase III‐I‐II cycle (i.e., interval between successive phase III contractions) were quantified. Thus, every observed phase III complex and MMC within a patient's ADM recording were counted and summated. Both antral and duodenal localizations were eligible for quantification. Lastly, the presence of abnormal neuropathic contraction patterns was assessed according to the following criteria^26^: (a) retrograde peristalsis, defined as either simultaneous or retrograde contractions over a segment >10 cm, (b) presence of bursts, defined as non‐propagated, high amplitude (>20 mmHg) and high frequency (10–12/min) phasic pressure activity for longer than 2 min; (c) clustered contractions for >20 min, defined as a pattern of 3–10 contractions within 5 s, followed by >1 min of absent motor activity. Neuropathic patterns were evaluated in both antrum and duodenum during the entire ADM recording (i.e., fasting and postprandial period).

### 2.4 Data analysis

All analyses were performed using IBM SPSS statistics for Windows, Version 25.0.^37^ Continuous variables are presented as means with standard deviation (SD) or as medians with interquartile ranges (IQRs) depending on normality of the distribution. Categorical variables were presented as counts with percentages. Two‐sided *p*‐values <0.05 were considered statistically significant. Fed period duration data analyses were performed using Kaplan–Meier statistics. Kaplan–Meier analysis is used to establish the fed period duration in the fraction of patients with observed phase III contractions for the etiology‐based subgroups. Cases with no observed phase III contractions were censored. Differences between the curves of the etiology‐based subgroups were calculated with the log rank test. A multivariable Cox regression model was used to adjust for differences in gender, age, BMI, clinical severity, and GE *T*_1/2_ as potential confounders. The number of MMCs and phase III contractions between GP subgroups was assessed using a generalized linear model (GLM) with log link fit for negative binomial distribution. The resulting exponentiated B (Exp B) is a ratio of arithmetic means, and thereby represents the multiplying factor between the different etiologies. To adjust for potential confounding, the previously mentioned variables were added to the model. The chi‐square Fisher's exact test was used to assess differences between GP subgroups regarding antral hypomotility, neuropathic patterns, and the absence of phase III contractions and MMCs. Post hoc analyses were performed to assess whether any of the observed motility patterns were associated with clinical severity. For sample size reasons, this was only performed in idiopathic GP patients. The main aim was to assess whether fed period duration, the number of MMCs and the number of phase III contractions were associated with clinical severity grade or GE *T*_1/2_. Based on the distribution within this present study, GE *T*_1/2_ was therefore classified into three categories: (1) GE *T*_1/2_<180 min; (2) GE *T*_1/2_181–250 min; and (3) GE *T*_1/2_ > 250 min. For fed period duration, the three clinical severity grades and three GE _T1/2_ groups were intermittently added as dummy variables in a multivariable Cox regression model to assess associations. For the count data (number of MMCs and phase III contractions), the dummy variables were intermittently added to a GLM with log link fit for negative binomial distribution. Both models were adjusted for potential confounding by previously mentioned variables.

## 3 RESULTS

### 3.1 Subjects

**FIGURE 1:**
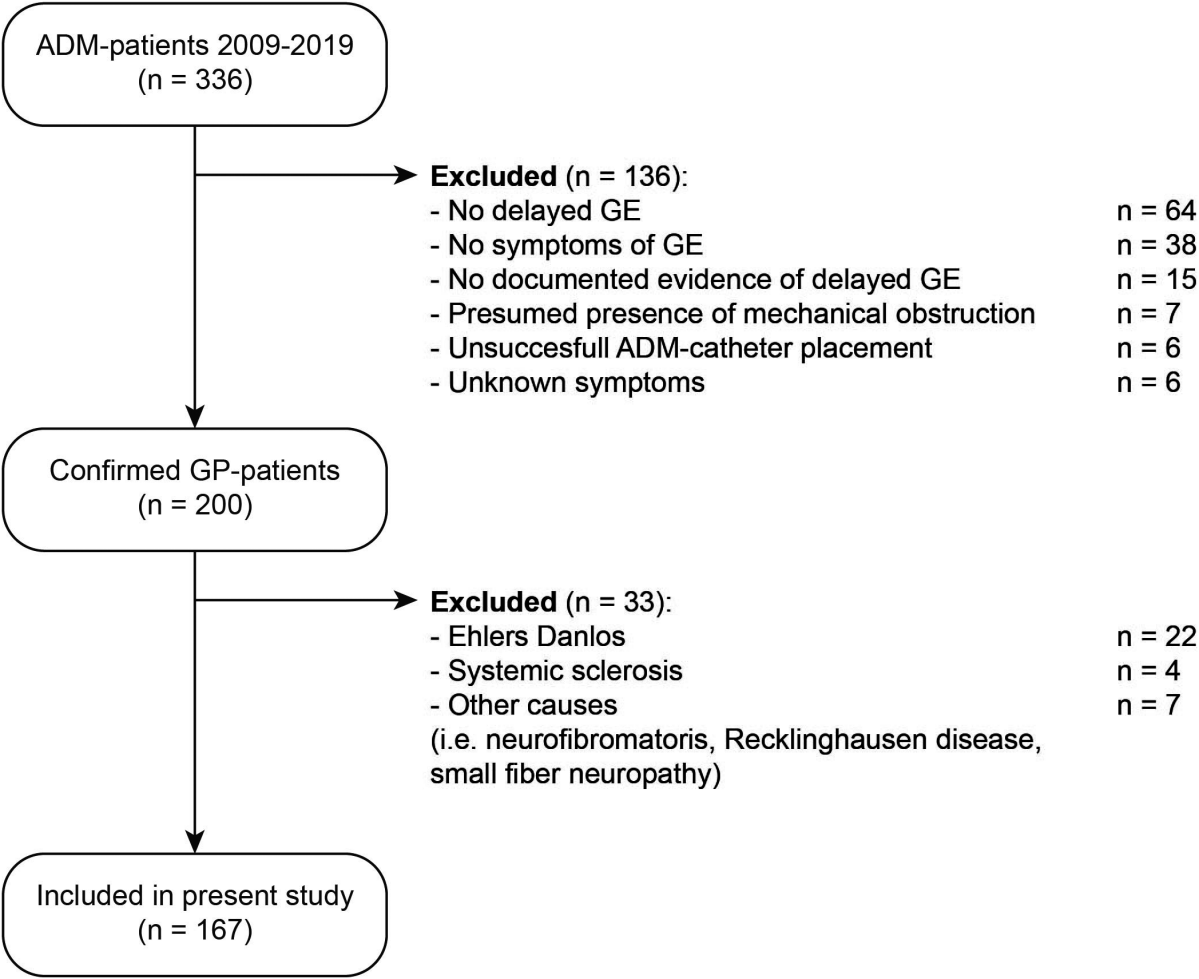
Flow chart of inclusion process. ADM, antroduodenal manometry; GE, gastric emptying; GP, gastroparesis; n, number of included patients

From 2009 to 2019, 336 patients underwent ADM for the investigation of enteric dysmotility at Maastricht University Medical Centre. After reviewing the medical records, 200 patients met the inclusion criteria (Figure 1). The other 136 patients were excluded for the following reasons: absence of delayed GE upon testing (*n* = 64), no symptoms of GP (*n* = 38), no documented evidence of delayed GE (*n* = 15), presumed mechanical obstruction (*n* = 7), unsuccessful ADM‐catheter placement (*n* = 6) or unknown symptoms (*n* = 6). Thirty‐three more patients were excluded as their sample sizes were too small for meaningful statistical analyses in distinct underlying etiologies (Ehlers–Danlos *n* = 22, systemic sclerosis *n* = 4, neurofibromatosis *n* = 3, small fiber neuropathy 3, and Recklingshausen disease *n* = 1). Baseline characteristics of the 167 enrolled patients with confirmed delayed GE are displayed in Table 1. The majority of GP patients was female, accounting for 92% of idiopathic, 86.7% of diabetic, and 63.9% of post‐surgery GP patients (*p* < 0.01). Median age and BMI were significantly lower in idiopathic GP patients compared with both post‐surgery and diabetic GP patients (*p* = 0.02 and *p* < 0.01 respectively). Clinical severity grades and GE *T*_1/2_ were equally distributed between the different etiologies of GP.

**TABLE 1:** Patient characteristics of included subjects with post‐surgery, idiopathic and diabetic gastroparesis

		Idiopathic (*n* = 101)	Post‐surgery (*n* = 36)	Diabetic (*n* = 30)	*p*‐value
Gender					
F	*N* (%)	93 (92.1)	23 (63.9)	26 (86.7)	<0.01^a^
M	*N* (%)	8 (7.9)	13 (36.1)	4 (13.3)	
Age	Mdn (IQR)	40 (28–55)	48 (35–68)	50 (42–56)	0.016^b^
BMI	Mdn (IQR)	21 (19–24)	24 (21–29)	26 (21–29)	<0.01^b^
GP Grade					
1	*N* (%)	16 (15.8)	4 (11.1)	1 (3.4)	0.194^a^
2	*N* (%)	33 (32.7)	10 (27.8)	6 (20.7)	
3	*N* (%)	52 (51.5)	22 (61.1)	22 (75.9)	
GE T_1/2_	Mdn (IQR)	176 (149–216)	190 (152–238)	193 (153–252)	0.236^b^

Abbreviations: BMI, body mass index in kg/m^2^; F, female; GE T1/2, gastric half emptying time in minutes; GP grade, clinical severity of gastroparesis; IQR, interquartile range; M, male; Mdn, Median; *n*, number of included patients; *N*, number of patients with variable present; *SD*, standard deviation.

^a^ Fisher’s exact test.

^b^ Kruskall–Wallis analysis.

### 3.2 Fed period duration

**FIGURE 2:**
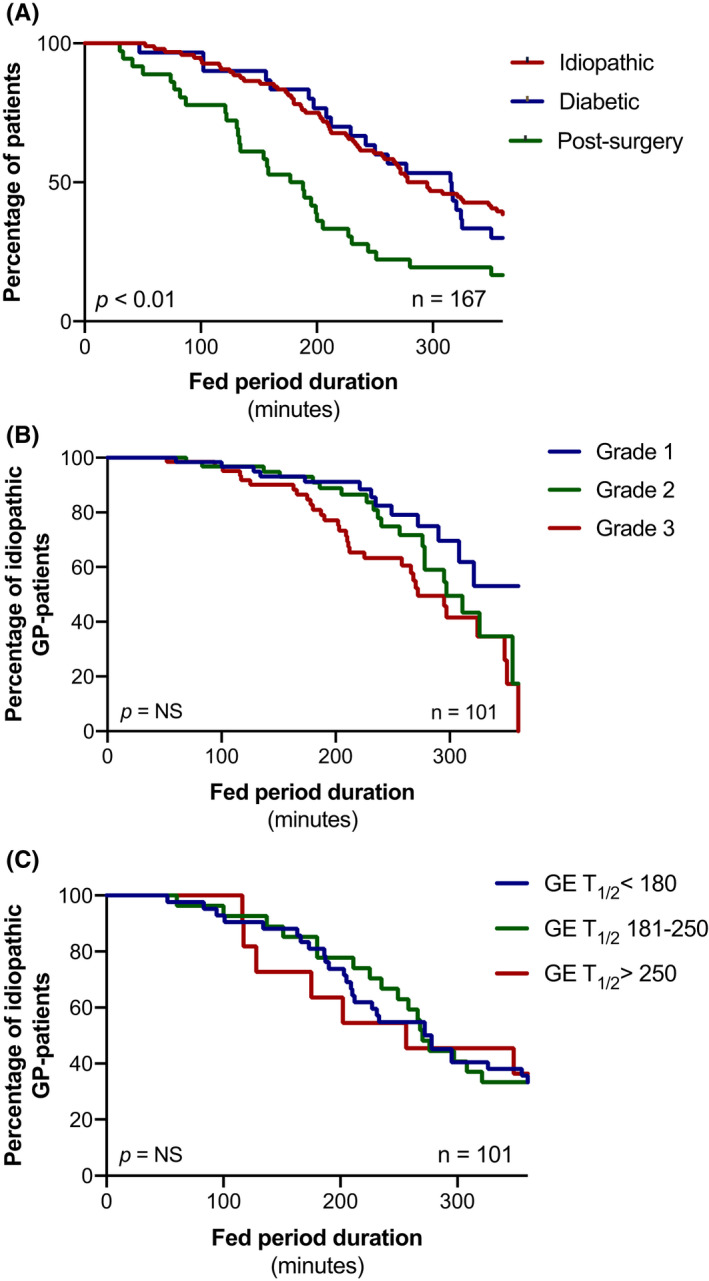
Kaplan–Meier curves of fed period duration (in minutes) in: (A) Post‐surgery, idiopathic, and diabetic gastroparesis patients. The *p*‐value represents the statistical difference between the curves which was calculated by log rank test and a multivariable Cox regression model to adjust for potential confounding by gender, age, BMI, clinical severity and gastric half emptying time (GE *T*_1/2_). (B) Idiopathic GP patients according to clinical severity grades of gastroparesis (defined as grade 1—mild symptoms, grade 2—moderate symptoms, grade 3—refractory symptoms); (C) Idiopathic GP patients according to severity of gastric half emptying time (defined as GE *T*_1/2_ < 180 min, GE *T*_1/2_ 181–250 min and GE *T*_1/2_ > 250 min)

Cox regression analysis showed that fed period duration was significantly different between GP subgroups (*p* < 0.05) (Figure 2). After adjustment for potential confounding, a longer fed period duration was observed in both idiopathic and diabetic GP patients compared with post‐surgery GP patients (*p* = 0.03; Table S1). No significant correlation was found between fed period duration and the clinical severity grades of GP or GE *T*_1/2_ groups within idiopathic GP patients (Figure 2). Likewise, the number of both phase III contractions and MMCs did not demonstrate a gradient to suggest a potential association with clinical severity grades or with GE *T*_1/2_ groups (Figure S1).

### 3.3 Number of MMCs and phase III contractions

**FIGURE 3:**
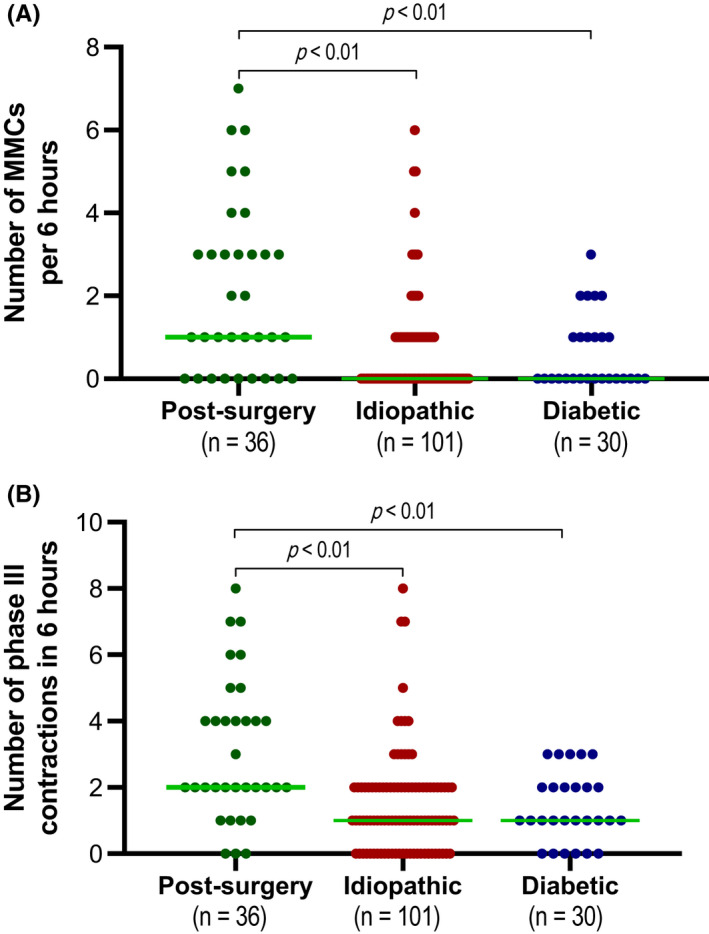
(A) Distribution of the observed number of migrating motor complexes (MMCs) during 6‐h antroduodenal manometry among post‐surgery, idiopathic, and diabetic gastroparesis patients. (B) Distribution of the observed number of migrating phase III contractions during 6‐h antroduodenal manometry among post‐surgery, idiopathic, and diabetic gastroparesis. Both figures correspond with count data. The green line represents the median number within each group. Adjusted *p*‐values were assessed using a generalized linear model with log link fit

**FIGURE 4:**
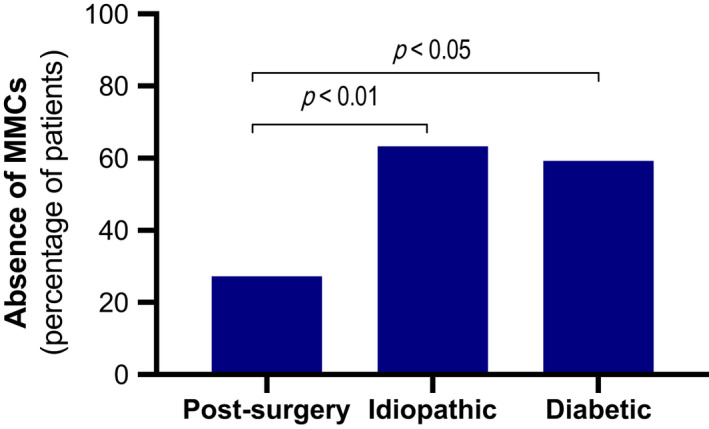
Proportional absence of migrating motor complexes (MMCs) for the percentage of patients within post‐surgery, idiopathic, and diabetic gastroparesis as assessed by chi‐square Fisher’s exact test

Significantly more MMCs and phase III contractions were observed in post‐surgery GP patients compared with the other etiologies (*p* < 0.01; Figure 3 and Table S2). Compared with post‐surgery GP patients, MMC numbers in idiopathic and diabetic GP patients were 3.6 and 4.8 times lower, respectively (Figure 3A). Moreover, a similar pattern was observed in the number of phase III contractions, that is, 2.3 and 3.1 times lower in idiopathic and diabetic GP patients, respectively (Figure 3B). In accordance, MMCs were more often absent in idiopathic (63.3%) and diabetic (59.3%) GP patients compared with post‐surgery GP patients (27.3%) (*p* < 0.05; Figure 4). Absence of phase III contractions was more often observed in idiopathic (27.6%) and diabetic (22.2%) compared with post‐surgery (9.1%), but not significantly different (*p* = 0.08). The number of MMCs or phase III contractions was not correlated with clinical severity grades or GE *T*_1/2_ (Figure S2).

### 3.4 Motility Index

**FIGURE 5:**
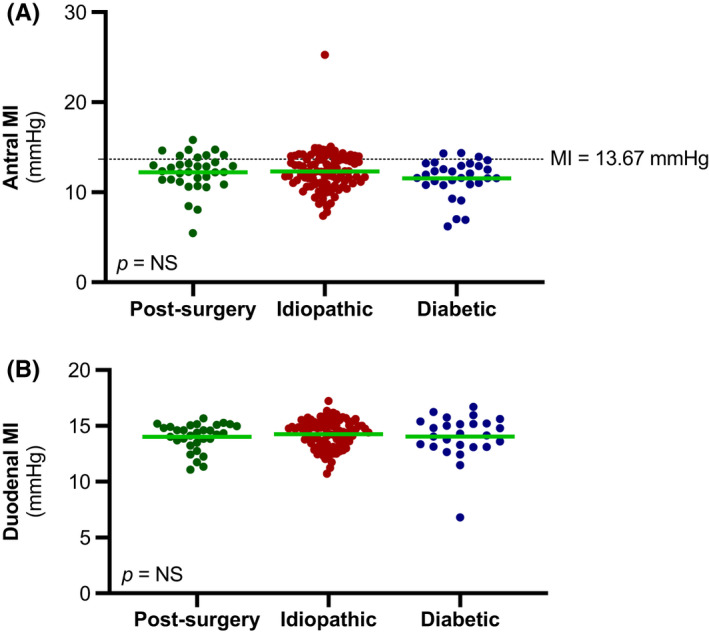
(A) Antral motility index (MI) of post‐surgery, idiopathic, and diabetic gastroparesis patients; (B) Duodenal motility index (MI) of post‐surgery, idiopathic, and diabetic gastroparesis patients. The green line represents the mean motility index within each group. The dotted line represents the cutoff point for antral hypomotility

Antral and duodenal MI were similar between diabetic, idiopathic, and post‐surgery GP patients (Figure 5). Antral hypomotility (i.e., MI <13.67 mmHg) was observed in 90% of diabetic GP patients compared with 75.2% and 76.5% of idiopathic and post‐surgery GP patients (*p* = 0.22). Duodenal origin of phase III contractions was observed in 67% of post‐surgery GP patients compared with 75% and 85% of idiopathic and diabetic GP patients. Antral MI was not correlated with clinical severity grades or GE *T*_1/2_.

### 3.5 Neuropathic patterns

Neuropathic patterns were observed in 69.4% of idiopathic, 66.7% of diabetic, and 64.5% of post‐surgery GP patients (*p* = NS, Table 2). Clustered contractions were observed in 5% of post‐surgery GP patients compared with 26.5% in idiopathic and 35.5% of diabetic GP patients (*p* = 0.05). Bursts and retrograde peristalsis were observed in respectively 42.5% and 39.6% of all GP patients, and no significant differences between etiologies were found.

**TABLE 2:** Number of patients with neuropathic patterns during 6–h antroduodenal manometry in post–surgery, idiopathic and diabetic gastroparesis patients

		Overall	Idiopathic (*n* = 98)	Post–surgery (*n* = 31)	Diabetic (*n* = 27)	*p*–value^a^
Neuropathic pattern	*N* (%)	106 (67.9)	68 (69.4)	20 (64.5)	18 (66.7)	0.855
Retrograde peristalsis	*N* (%)	42 (39.6)	22 (32.4)	11 (55.5)	9 (50.0)	0.127
Bursts	*N* (%)	45 (42.5)	28 (41.2)	8 (40.0)	7 (41.2)	0.995
Clustered contractions	*N* (%)	26 (24.5)	18 (26.5)	1 (5.0)	6 (35.3)	0.053

Abbreviations: *N*, number of patients with presence of the neuropathic pattern; *n*, number of valid patients within each group.

^a^ Fisher’s exact test.

## 4 DISCUSSION

In the present study, antroduodenal motility patterns were compared among gastroparesis patients with different etiologies. The main findings are as follows: (1) fed period duration was significantly longer in idiopathic and diabetic GP patients compared with post‐surgery GP patients; (2) number of both phase III contractions and MMCs was significantly lower in these groups compared with post‐surgery GP patients; (3) correspondingly, diabetic and idiopathic GP patients significantly more often presented without any MMC during 6 h of recording compared with post‐surgery GP patients. Taken together, the outcomes of the present study indicate that antroduodenal motility patterns are different among GP etiologies.

The underlying pathophysiological mechanisms of GP remains poorly understood. The most widely accepted hypothesis is that diabetic and idiopathic GP are caused by intrinsic neuropathy (i.e., immune‐modulated loss of pacemaker ICCs), whereas in post‐surgery, it is thought to be caused by extrinsic neuropathy (i.e., vagus nerve damage). In diabetic and idiopathic GP patients, loss of ICCs is the most reported cellular abnormality, with the deficiency being more prominent in the gastric antrum than in the body.^19^, ^22^, ^23^, ^24^, ^38^ Loss of ICCs has been associated with gastric dysrhythmias in diabetic GP patients, such as loss of slow‐wave generation or re‐entry tachycardias.^21^, ^39^ Furthermore, loss of ICCs correlated with the delay in GE in diabetic GP patients. Consequently, antral ICC deficiency is suggested to impair slow‐wave generation and electrical conduction, thereby interfering with enteric coordination, and causing delayed GE. The dysmotility spectrum identified in the present study supports this hypothesis, as different dysmotility patterns were observed in GP patients with assumed intrinsic neuropathy. It is conceivable that antral ICC deficiency impairs the slow‐wave basal electrical rhythm, thereby impairing the subsequent generation of phase III contractions and MMCs as observed in the present study, and resulting in delayed GE. Another possible explanation is absence of neuroendocrine signals that modulate GE based on the composition of the chyme, or dysfunctional antro‐pyloro coordination, in particular with relation to the relaxation of the pylorus, which is necessary to empty the stomach.^8^ Two prior studies reported pylorospasm in diabetic GP patients.^40^, ^41^ Unfortunately, assessing pyloric activity by ADM has proven technically difficult to reproduce due to inaccuracy of the sensors output at low amplitudes and catheter migration during peristalsis.^42^ Therefore, measuring pyloric activity was not feasible in the present study.

To the best of our knowledge, no prior studies have compared antroduodenal motility patterns among different etiologies of GP. The findings in post‐surgery gastroparesis fit well with the typical findings observed in patients with vagal nerve injury and could explain the differences found with diabetic and idiopathic gastroparesis. Typically, these studies reported increased number of MMCs and antral hypomotility as seen in the post‐surgery group of the present study.^29^, ^43^, ^44^ Antral hypomotility has long been suggested as the major cause of delayed GE in diabetic gastroparesis.^45^ In the present study, antral hypomotility was observed in 90% of diabetic GP patients compared to 75.2% and 76.5% of idiopathic and post‐surgery GP patients, although this difference was not significant. A recent study by Cogliandro et al. reported a high prevalence of duodenal dysmotility in patients with GP symptoms as measured by duodenal manometry.^26^ The same study evaluated duodenal motility according to definitions of neuropathic patterns similar to the ones used in the present study. Neuropathic patterns were comparable in both studies as follows: retrograde peristalsis was 43.2% compared to 39.6% in this study; bursts as 54.5% and 42.5%; and clustered contractions as 35.2% and 24.5%, respectively. Hypomotility and absence of phase III contractions were less often observed in Cogliandro's study. It is worth mentioning that the majority of the included subjects in their study had no delayed GE, and motility was measured in the duodenum exclusively. Lastly, it was reported that presence of duodenal dysmotility was more strongly associated with the degree of clinical severity than GE. This is in line with the poor association of GE with symptom severity reported in literature^25^ and suggest that we ought to shift our focus to alterations in duodenal, or more specifically antroduodenal, motility in GP. The exact relation to symptom severity and such motility patterns, however, remains to be established.

Antroduodenal manometry is a complex and time‐consuming method used in tertiary referral centers to evaluate gastric and duodenal coordination and activity by recording the overall nature of motility.^27^ The use and interpretation of ADM yield multiple challenges as no standard protocols are available with respect to study duration and meal composition. Furthermore, normal values on the different ADM parameters are still lacking. Studies are awaited to establish the viability of ADM by formulating standardized ADM protocols and normal values. Pyloric activity should also be considered in these studies.

Strength of the present study is the unprecedented comparison of motility patterns simultaneously registered in both the antrum and the duodenum across different GP etiologies within this rather large sample size cohort. ADM data were collected prospectively according to a standardized protocol in a cohort of gastroparesis patients. Furthermore, a solid‐state high‐resolution antroduodenal catheter was used, which is considered the gold standard for measuring antroduodenal motility.^35^ The definition of antral hypomotility was obtained from a previous study using water‐perfused catheters. Water‐perfused catheters detect overall lower amplitudes than the solid‐state catheters used in the present study. Therefore, an overall underestimation of antral hypomotility could be expected in the present study. However, this does not interfere in comparing the etiology‐based subgroups. The main limitation of this study is the absence of a control group with normal GE. Furthermore, baseline GE tests were performed in multiple referring centers using different protocols, being a possible confounder in the data analysis. Scintigraphy is currently considered the gold standard, but it involves radiation exposure and is expensive.^35^ ^13^C breath test has been recognized as a reasonable alternative for GE testing.^30^, ^46^ In the present study, scintigraphy and ^13^C breath test have been merged to one variable by using the coefficient 0.89, which was derived from the main validation study on the ^13^C breath test.^30^ This enabled comparison with GE severity, yet introduced some inaccuracy. Since the present study covers a period of 10 years, no systematic symptom severity scores were registered. It would be interesting to correlate the present results with symptom severity, mainly as the GE tests lack correlation with symptom severity, while enteric dysmotility was suggested to be better correlated.^25^, ^26^ Instead, the present study assessed the association of abnormal motility patterns to the clinical severity grades of GP.^3^

The outcomes of the present study can potentially aid in further refinement of diagnostic workup and more accurate targeted treatment options. In a recent study, we performed a comprehensive motility analysis in patients with decompensated GP undergoing gastric peroral endoscopic pyloromyotomy (G‐POEM).^47^ In a relatively small group of GP patients, no potential predictors of clinical response after G‐POEM could be identified among ADM and pyloric distensibility parameters. Sleeve gastrectomy has also been suggested as surgical treatment option to improve quality of life for selected patients with GP.^48^ ADM might be a helpful diagnostic method to predict clinical outcome of these potential treatment options, thereby enabling a more targeted treatment approach.

In summary, we present a comprehensive antroduodenal motility analysis in GP patients showing that antroduodenal motility patterns are different among GP etiologies. A dysmotility spectrum was identified with different patterns ranging from post‐surgery GP to idiopathic and diabetic GP. Case–control studies with GP patients of varying etiology with the use of validated patient‐rated symptom assessment (e.g., gastroparesis cardinal symptom index^49^) are warranted. This can lead to a better understanding of different pathophysiologic pathways and possible customized treatment options among GP etiologies.

## AUTHOR CONTRIBUTIONS

M.J.M. Hereijgers and J.M. Conchillo contributed to study concepts and designs, data analysis, and interpretation and drafting of the manuscript. All other authors critically revised the manuscript and approved the final copy.

## CONFLICT OF INTEREST

All authors declare no conflict of interests.

## Supporting information

Supplementary Material
