# Supplementary material for: Antroduodenal motility recording identifies characteristic patterns in gastroparesis related to underlying etiology
Source: Neurogastroenterol Motil. 2022 May 9;34(11):e14394. doi: 10.1111/nmo.14394 (PMC9788094; doi:10.1111/nmo.14394)
Supplement: Supplementary file 1 — Supplementary Material [file NMO-34-e14394-s001.docx]

Supporting Information

**Supplemental Table 1.** Comparison of fed duration period among idiopathic, diabetic and post-surgery GP-patients using a multivariable Cox’s regression model. Differences in hazard ratios between subgroups are expressed as adjusted odds ratios (OR) with accompanying 95% confidence intervals (95% CIs) and adjusted *p*-values (adj. *p*).

| **Fed period duration** (minutes) | **Unadjusted**^†^ | | |  | **Adjusted**^‡^ | | |
| --- | --- | --- | --- | --- | --- | --- | --- |
|  | OR | 95% CI | *p*-value |  | OR | 95% CI | *p*-value |
| Idiopathic vs. Post-surgery | 2.0 | 1.2 – 3.3 | 0.011 |  | 1.9 | 1.1 – 3.4 | 0.028 |
| Idiopathic vs. Diabetic | 0.9 | 0.5 – 1.7 | 0.695 |  | 0.9 | 0.4 – 1.7 | 0.850 |
| Post-surgery vs. Diabetic | 0.5 | 0.2 – 0.9 | 0.030 |  | 0.5 | 0.2 – 0.9 | 0.034 |

OR: Odds ratio; 95% CI: 95 percent confidence interval.

^†^ Unadjusted: multivariable Cox regression model without adjustment for potential confounding.

^‡^ Adjusted: multivariable Cox regression model with adjustment for gender, age, body mass index, gastric half emptying time and grade of gastroparesis.

**Supplemental Table 2.** Comparison of number of MMCs and phase III contractions during 6-hours antroduodenal manometry among idiopathic, diabetic and post-surgery GP-patients as measured by a generalized linear model with log link fit. Differences in Exp Bs between subgroups are expressed as (un)adjusted Exp Bs with accompanying 95% CIs and p-values.

| Variable | **Unadjusted**^†^ | | |  | **Adjusted**^‡^ | | |
| --- | --- | --- | --- | --- | --- | --- | --- |
|  | Exp. B | 95% CI | *p*-value |  | Exp. B | 95% CI | *p*-value |
| **Number of MMCs** |  |  |  |  |  |  |  |
| Idiopathic vs. Post-surgery | 3.2 | 2.0 – 5.2 | <0.01 |  | 3.6 | 2.0 – 6.5 | <0.01 |
| Idiopathic vs. Diabetic | 0.9 | 0.5 – 1.8 | 0.871 |  | 0.7 | 0.3 – 1.6 | 0.469 |
| Diabetic vs. Post-surgery | 3.4 | 1.8 – 6.2 | <0.01 |  | 4.8 | 2.3 – 10.4 | <0.01 |
| **Number of phase III contractions** | |  |  |  |  |  |  |
| Idiopathic vs. Post-surgery | 2.0 | 1.5 – 2.8 | <0.01 |  | 2.3 | 1.6 – 3.4 | <0.01 |
| Idiopathic vs. Diabetic | 1.1 | 1.0 – 1.8 | 0.637 |  | 1.4 | 0.8 – 2.2 | 0.224 |
| Diabetic vs. Post-surgery | 2.2 | 1.6 – 3.2 | <0.01 |  | 3.1 | 1.9 – 5.2 | <0.01 |

Exp. B: exponentiated coefficient B^§^; 95% CI: 95 percent confidence interval; MMC: migrating motor complex.

^†^Unadjusted: assessed by generalized linear model with log link fit for negative binomial distribution, without adjustment for potential confounding.

^‡^ Adjusted: assessed by generalized linear model with log link fit for negative binomial distribution, with adjustment for gender, age, body mass index, gastric half emptying time and grade of gastroparesis.

^§^ Exponentiated coefficient B is a ratio of arithmetic means and thereby represents the multiplying factor between the compared two etiology-based subgroups.

**Supplemental Figure 1.** Illustrative figure of a typical high-resolution antroduodenal manometry recording.

**Supplemental Figure 2.** Mean number of observed migrating motor complexes (A) and phase III contractions (C) in idiopathic GP-patients according to clinical severity grades of gastroparesis (defined as: grade 1 - mild symptoms, grade 2 - moderate symptoms, grade 3 - refractory symptoms). Mean number of observed migrating motor complexes (B) and phase III contractions (D) in idiopathic GP-patients according to severity of gastric half emptying time (GE T1/2) in minutes defined as: GE T_1/2_ < 180 minutes, GE T_1/2_ 181-250 minutes and GE T_1/2_ >250 minutes).
